# Emerging Trends and Future Directions for the Field of Motivation Psychology: A Special Issue in Honor of Prof. Dr. Willy Lens

**DOI:** 10.5334/pb.354

**Published:** 2016-07-13

**Authors:** Maarten Vansteenkiste, Athanasios Mouratidis

**Affiliations:** 1 University of Gent, Belgium; 2 Hacepette University, Turkey

## Abstract

This special issue is devoted to Prof. Dr. W. Lens, who passed away end of August 2014 while he was vacationing. The special issue is meant to honor Willy’s intellectual contribution to the field of motivation psychology and his enthusiastic and devoted mentorship, which has spurred many of us to study motivation-related topics. In line with Willy’s interest and extensive network, the special issue brings together scholars from diverse theoretical perspectives (i.e., Achievement Goal Theory, Future Time Perspective Theory, and Self-Determination Theory) and diverse cultural backgrounds (i.e., China, Peru, Greece, Portugal, Belgium, US, Australia, Canada). We introduce the special issue by highlighting four emerging trends that characterize contemporary motivation psychology and that were central to Willy’s work: (a) multiperspectivism (i.e., a reliance on multiple motivational frameworks); (b) the diversity of motives and goals that underlie behavior (i.e., motivational heterogeneity); (c) interest in motives for non-participation; and (d) the issue of universalism versus relativism (i.e., the question whether there exist universal motivational processes or whether these processes are contingent upon sociodemographic, personality-based, and contextual factors). Each of the eight contributions in the special issue touch upon one or more of these emerging themes, which are critically discussed in conjunction with a number of directions for future research.

## Publisher's Note

This article was originally published with the incorrect pagination: 118‑142. This error has now been fixed.

## Article

This special issue is devoted to Prof. Dr. Willy Lens, who unexpectedly passed away while vacationing at the end of August 2014. The contributors of this special issue aim to honor Willy Lens intellectual heritage and to commemorate his sincere interest in the field of motivation psychology. Willy was known for his contagious enthusiasm and curiosity. He has stimulated many scholars across the world to pursue motivation-related topics, some of whom contributing to this special issue. Central to the field of motivation psychology, but also to Willy’s career, were the questions why people engage in an activity and which factors energize people’s engagement and continued persistence. Being trained in the tradition of achievement motivation theory (e.g., Atkinson, 1964; Atkinson & Feather, 1966), Willy broadened his theoretical scope throughout his career, thereby doing research from a variety of theoretical frameworks, including Expectancy-Valence Models (e.g., Van den Broeck, Vansteenkiste, Lens, & De Witte, 2010; Wigfield, Tonks, & Klauda, 2009), Achievement Goal Theory (e.g., Maehr & Zusho, 2009; Matos, Lens, & Vansteenkiste, 2009; Senko, Hulleman, & Harackiewicz, 2011), and Self-Determination Theory (Deci & Ryan, 2008). Yet, his most favorite topic involved the question to what extent the depth of people’s future time perspective (e.g., Husman & Lens, 1999; Simons, Vansteenkiste, Lens, & Lacante, 2004) as well as their attitude (i.e., positive or negative) towards the future (Devolder & Lens, 1982) impact on their present goal setting and striving (Lens, Simons, & Dewitte, 2002), their motivation (Mouratidis & Lens, 2015) and, more broadly, their identity development (Luyckx, Lens, Smits, & Goossens, 2010).

As Willy progressed through his career, he witnessed an increasing interest in the topic of motivation, both among academics and practitioners. The field of motivation psychology has burgeoned over the past two decades, as illustrated by the publication of an increasing number of handbooks on motivational topics, some with a strong theoretical focus (e.g., Ryan, 2012b; Shah & Gardner, 2008) and others with a more applied focus in domains as diverse as education (e.g., Wentzel, Wigfield, & Miele, 2009), sports (Roberts & Treasure, 2012), health care (Rollnick, Miller, & Butler, 2008), and psychotherapy (Michalak & Holtforth, 2006).

One of the reasons for this exponential increase in interest for motivation is the fact that motivation research has direct applied value (Ryan, 2012a). This applied value is captured in Kurt Lewin’s quote that “there is nothing as practical as a good theory”, a quote that Willy posted on the door of his office. Many socializing agents in real life, including parents, teachers, sport coaches, doctors, or managers, are facing the challenge to motivate people to carry out requests, to make a change, or to actualize their potential. Political leaders also have a motivational role, for instance to develop policies to activate unemployed people to find a job, to stimulate obese people for adopting a more healthy lifestyle, or to stimulate immigrants to acquire the language of the immigration country. Although there is often a consensus among politicians *that* these more vulnerable groups need to be activated or motivated, the *way* how this is done varies widely across political parties and ideologies. For instance, depending on one’s political affiliation, an activation policy for the unemployed can take the form of being highly prescriptive and controlling or can be rather supportive, thereby better taking into account people’s rhythm, situation, and personal choices. Note that motivation is not only relevant in a vertical and hierarchically structured relationships but also in more horizontal relationships, including colleagues at work (Moreau & Mageau, 2012), teammates in sport clubs (Fransen et al., 2015), and siblings at home (Van der Kaap-Deeder, Vansteenkiste, Soenens, & Mabbe, 2016).

In the following paragraphs, we introduce the eight contributions that are part of the special issue by indicating how each of them reflect Willy’s interests and how they are indicative of broader themes and trends that can be observed in the motivation literature. For each of the four discussed trends, we offer a number of theoretical reflections, which are in many cases also inspired by the lively and sometimes heated discussions we had with Willy. The following trends are discussed: (a) the evolution from a mono-theoretical to a multi-theoretical motivational perspective; (b) an increasing emphasis on motivational heterogeneity; (c) a tendency to identify both reasons for both partaking and not partaking in an activity; and (d) an increasing interest in the question whether certain motivational pathways and approaches are universally applicable or instead depend on various factors that qualify their effects (e.g., sociodemographic, cultural, and personality-related variables). Some of these themes have been around in the literature for quite some time, but were rather dormant and are enjoying increasing interest today. Of course, we could have chosen other topics and trends (e.g., an increasing focus on intervention work; the investigation of biological and neurological correlates of motivational functioning, see Ryan, 2012b), but our choice reflects Willy’s interests as well as the contributions in this special issue.

### Trend 1: The Evolution from a mono-theoretical to a multi-theoretical motivational perspective

**Theoretical Richness of the Field.** Traditionally, the field of motivation psychology is theoretically rich. During the past century, a variety of frameworks were developed, with some of them having a more cognitive focus – e.g., Social Cognitive Theory (Bandura & McClelland, 1977); Attribution Theory (Weiner, 1985); Theory of Planned Behavior (Ajzen, 1991) – and with others focusing more on experiential and affective features of people’s motivations, such as experiences related to critical psychological needs – e.g., Self-Worth Theory (Covington, 1992) or Self-Determination Theory (Deci & Ryan, 2000).

One trend that characterizes contemporary motivation psychology over the last two decades is an increasing reliance on multiple top-down theoretical perspectives or bottom-up models that have grown out of practice (see also Ford & Smith, 2009). Rather than working from a single theory, scholars quite often combine different theories into a more *encompassing* framework or model (e.g., Chan et al., 2015; Janke, Nitsche, & Dickhäuser, 2015), which is often depicted as being more integrative. This trend was visible also in Willy’s own work, as he sought to approach individuals’ (lack of) motivation from diverse theoretical angles, an approach which is often refreshing, illuminating, and enriching. Willy argued that through the development of a more encompassing framework, the similarities and differences between different motivational frameworks and notions may get clarified. Such conceptual and operational clarification may then possibly lead to a reduction of the amount of motivational vocabulary (and measures) in the field. Indeed, newcomers in the field may easily get overwhelmed by the number of frameworks, concepts, and measures. As scholars, we thus face the challenge to distinguish essential from superficial distinctions, both at the conceptual level and at the level of assessment (e.g., Hulleman, Shrager, Bodmann, & Harackiewicz, 2010), as to achieve a higher level of parsimony. This task is critical to facilitate progress in the academic field but also to improve effective communication of science to practice.

At the same time, we hasten to add that the mere linkage of motivational concepts from diverse theories in an empirical contribution does not imply that these theories get integrated or unified in a deep and fundamental way. Toward this end, we advocate a thoughtful and selective use of the term ‘integration’. To illustrate, the empirical observation that mastery-oriented students report more autonomous or volitional motives for their school work, while performance-oriented students report more controlled or pressured motives (Su, McBride, & Xiang, 2015) does not mean by itself that Achievement Goal Theory and Self-Determination Theory get integrated. Measures tapping into core concepts of both frameworks have merely been linked at the empirical level (e.g., Ciani, Sheldon, Hilpert, & Easter, 2011). If the mere empirical linkage of measures from diverse theoretical frameworks does not suffice for a meaningful theoretical integration, which conditions are required to integrate theories more fully?

**Critical Conditions for Theoretical Integration.** Three conditions seem critical. First, a compelling theoretical necessity is warranted. That is, different frameworks need to be *complementary*, thereby compensating each other’s weaknesses by their respective strengths. For instance, fully developed motivation theories need to address both the direction people move toward as well as the factors that energize people’s motivational functioning (Deci & Ryan, 1985). The development of the hierarchical model of achievement motivation brought the classic work on achievement motives (pertaining to the energization of behavior) together with the more recent work on achievement goals (pertaining to the direction of behavior) in a truly integrated fashion (Elliot, 1999). In other cases, motivational models or approaches – which were often developed out of practice – have been combined in a complementary way with top-down theories that shed light on more fundamental motivational processes. To illustrate, while Motivational Interviewing (Miller & Rollnick, 2012) provides insights in how exactly clinicians can motivate clients who display resistance (‘how’-component), the processes activated by a motivational interviewing style can be understood on the basis of Self-Determination Theory (‘why’-component; Markland, Ryan, Tobin, & Rollnick, 2005; Vansteenkiste & Sheldon, 2006; Vansteenkiste, Williams, & Resnicow, 2012).

Second, to proceed toward theoretical integration, the *clarification* of the theoretical and operational boundaries of core concepts of different frameworks is required, a point that Willy repeatedly emphasized during his career. As an illustration, scholars in Expectancy-Valence Theory (Eccles & Wigfield, 2002) have emphasized the fact that tasks need to contain high perceived utility value or usefulness to be motivating (e.g., Harackiewicz, Rozek, Hulleman, & Hyde, 2012; Husman, Hilpert & Brem, this issue). Similarly, from the perspective of Self-Determination Theory, a meaningful rationale needs to be given for an assigned task such that people come to identify with the personal relevance of the behavior, which eventually promotes a more volitional engagement (Deci, Eghrari, Patrick, & Leone, 1994; Jang, 2008). However, tasks may have high perceived utility value, without necessarily promoting a self-endorsed (i.e., volitional) engagement. Take the example of a child making homework in order to be allowed by its parents to watch television. While doing the homework would be instrumental to watch television and, hence, carry high utility value in the eyes of the child, the child would not necessarily have internalized the personal value of making homework. To fully endorse the reason for making homework, a child needs to understand how the homework carries some personal significance, for instance, because it is congruent with a personally held value or goal. This example illustrates that although key concepts of different frameworks seem similar at the surface, closer conceptual scrutiny reveals that at a deeper level there might be substantial differences. The challenge for motivation psychologists is to infer and ideally propose hypotheses to delineate when these similarities and differences should result in converging or diverging predictions, which can be tested in research.

A third condition for theoretical unification requires a clarification of the *meta-theoretical foundation* underlying several theories. For two theories to be deeply integrated, they need to be rooted in a similar view on human nature. This will require scholars to grabble with fundamental questions (Deci & Vansteenkiste, 2004), such as the question whether individuals are naturally pro-active, thereby taking initiative and being self-directed or whether, instead, they are better portrayed as being passive or reactive, only being pushed into activity via external contingencies. Without considering the main assumptions underlying a theory, the mere connection of different frameworks at the empirical level (i.e., in a particular study) has the risk of resulting in an epistemologically fragmented and even inconsistent approach that makes a minimal contribution at the theoretical level, while adding unnecessary complexity at the empirical level.

**This issue.** Reflecting this trend toward multiperspectivism, several contributions aim to bring together two frameworks at the empirical level. For example, Fryer, Van den Broeck, Ginns, and Nakao (this issue) sought to combine insights from Future Time Perspective Theory, which emphasizes the importance of individuals’ orientation toward distant and future goals instead of immediate goals (Lens et al., 2002), and insights from Self-Determination Theory, which highlights the quality of individuals’ motivational regulation, which can be either more pressured or more volitional in nature (Deci & Ryan, 2000).

Three other contributions (i.e., Delrue, Mouratidis, Muynck, Aelterman, & Vansteenkiste, this issue; Gaudreau & Braaten, in press; Michou, Matos, Gargurevich, Herrera, & Gumus, this issue) focus on the interplay between Achievement Goal Theory and Self-Determination Theory. Achievement goals were initially defined rather broadly (e.g., Maehr & Zusho, 2009; Nicholls, 1984; Senko et al., 2011), thereby encompassing a combination of both the type of aim learners set for themselves in achievement settings and a reason for pursuing such goals. Yet, Elliot (2005) suggested narrowing the definition of achievement goals to aims only (see also Senko, 2016). For example, learners could be focused on mastering the requirements of the task at hand (i.e., mastery goal) or outperforming others on a test (i.e., performance goal), yet, their reasons for pursuing each of these goals could vary. The conceptual detachment of reasons and aims paved the way for a systematic study of a variety of reasons underlying achievement goals (e.g., Dompnier et al., 2015; Urdan & Mestas, 2006). Vansteenkiste, Lens, Elliot, Soenens, and Mouratidis (2014) argued that Self-Determination Theory was ideally suited to fill this *conceptual vacuum*, as SDT-scholars have a long tradition in studying the reasons underlying individuals’ activity engagement (Ryan & Connell, 1989) and goal pursuit (Sheldon, 2002). Heeding this call to study both the “what” and “why” of achievement goals, three studies in this issue investigate the autonomous or volitional and controlled or pressured reasons underlying intra-personal goals (Delrue et al., this issue), mastery-approach and mastery–avoidance goals (Michou et al., this issue) and mastery-approach and performance-approach goals (Gaudreau & Braaten, , this issue). Across these studies, findings demonstrate that these reasons do matter to predict individuals’ functioning in achievement settings above and beyond the endorsement of achievement goals per se. In some cases, the “what” and “why” of achievement goals also interacted with each other to predict outcomes not accounted for by either of them separately (see also Gaudreau, 2012). Presumably, the goal complex (Elliot, 2006), that is, the presence of a specific achievement goal in combination with a specific reason, alters the meaning of the goal, thereby resulting in different outcomes.

Overall, these studies illustrate that the combination of frameworks can generate new practically useful insights. That is, while the pursuit of mastery goals was generally deemed to be adaptive from the normative goal perspective (Maehr & Zusho, 2009; Midgley, Kaplan, & Middleton, 2001), it appears that being focused on mastering the task at hand does not come with the same benefits when individuals stand under pressure to pursue this goal (Benita, Roth, & Deci, 2014). Conversely, the pursuit of performance goals does not necessarily yield a cost. When the goal of outperforming others is seen as a challenge to which one is committed (i.e., autonomously motivated) rather than as means to prove one’s worth and validate one’s ego (i.e., controlled motivated), these same performance goals yield different outcomes. This observation is perhaps especially illuminating for the field of sports, as performance goals are almost inherently connected to the competitive nature of sports.

**Summary.** With the exponentially growing interest in the topic of motivation, scholars have sought to combine different motivational theories or models, thereby developing more encompassing frameworks. In this special issue, several authors have relied on concepts from more than one motivational framework. Although such multi-theoretical studies are informative, it remains to be seen whether such a more encompassing framework advances the field over a longer period of time. In our view, for sustainable progress to be made, it is important to further pursue a conceptual analysis of the compatibility of the meta-theoretical assumptions behind the combined frameworks and a more precise articulation of conceptual (dis)similarities.

### Trend 2: An Increasing Focus on Motivational Heterogeneity

**From Unidimensional to Multidimensional Viewpoints.** Over the past decades, it has become increasingly clear that individuals’ motivation cannot be treated exclusively in a quantitative way, as if individuals would differ only in the *amount* or *dose* of motivation they display. As a result, a major breakthrough in the literature was that individuals’ motivation differs substantially as a function of the presence of qualitatively different dimensions of motivation (Deci & Ryan, 1985; Lens & Vansteenkiste, 2006; Vansteenkiste, Lens, & Deci, 2006). Even when individuals are equally motivated in quantitative terms, individuals can display different dimensions of motivation, with one dimension being of a higher *quality* and more desirable than the other. Depending on the guiding theoretical framework, individuals’ quality of motivation has been depicted as being intrinsically or extrinsically motivated (Harter, 1981), task- or performance-oriented (Nicholls, 1984) and autonomously or controlled motivated (Deci & Ryan, 2000).

The next challenge involved sorting out how these different dimensions of motivation would relate to each other. In some frameworks, individuals’ quality of motivation was conceived as falling along a single continuum, with the one pole representing the least desirable dimension of motivation and with the other pole representing the most desirable dimension of motivation. Underlying such a unidimensional view on individuals’ quality of motivation was the notion that different dimensions of motivation cannot *coexist*: the presence of poor quality motivation would preclude the presence of good quality motivation, while the presence of good quality motivation would represent a buffer against the emergence of poor quality motivation. At the operational level, such a unidimensional view was apparent in the development and use of unidimensional motivation scales (e.g., see Lepper, Corpus, & Iyengar, 2005 about the Harter’s scale), which requested participants to weight different dimensions of motivation against each other.^1^

**Motivational Profiles.** Yet, it became increasingly clear that motivational quality is not a black or white matter. Instead, it is matter of gradation, as different dimensions of motivation can easily coexist within a single person. To illustrate, an athlete can enjoy experimenting with a new technique and at the same time aim to prove to others that he is capable of mastering the technique. Because our behavior is by definition multi-determined, different dimensions of motivation need to be assessed separately rather than being pitted against each other (see Lepper et al., 2005). These separate motivational dimensions can then be organized into *motivational profiles* or *types* that capture naturally occurring combinations of types of motivation within subgroups of individuals. The topic of motivational heterogeneity or pluralism (Ford & Smith, 2009) received increasing empirical attention over the past decade across different life domains, including education (Ratelle, Guay, Vallerand, Larose, & Senecal, 2007; Tsoi, de Boer, Croiset, Koster, & Kusurkar, 2016), physical education (Aelterman, Vansteenkiste, Soenens, & Haerens, 2016; Haerens, Kirk, Cardon, De Bourdeaudhuij, & Vansteenkiste, 2010), sports (Gillet, Vallerand, & Rosnet, 2009), and work (Van den Broeck, Lens, De Witte, & Van Coillie, 2013) as well as across different theoretical frameworks, such as Achievement Goal Theory (e.g., Jang & Liu, 2012; John Wang, Biddle, & Elliot, 2007), Self-Determination Theory (e.g., Vansteenkiste, Sierens, Soenens, Luyckx, & Lens, 2009) or a combination of frameworks (Chian & Wang, 2008).

To shed light on different motivational profiles, scholars have typically used more inductive and person-centered analyses (e.g., cluster analysis and latent profile analysis) instead of variable-centered analyses. The number and type of motivational profiles obtained has varied across investigations. This is probably due to the variation across studies in the number of motivational dimensions included to create and characterize each motivational profile as well as the age groups and contexts being studied.

We believe this topic deserves greater attention in future work as human behavior is *multi-determined*, a point of view that Willy advocated wholeheartedly. In this respect, he often provided anecdotal examples indicating how the presence of controlled or pressuring motives next to autonomous motives may not be entirely harmful, an issue he sought to pursue with his students (Van den Broeck et al., 2013; Vansteenkiste et al., 2009). In addressing this issue of *motivational heterogeneity* within the person-centered approach, we would like to highlight a couple of issues.

First, we would like to warn for a potential problem of reification. On the basis of person-centered analyses it is tempting to conclude that motivational profiles are as real and distinct as groups distinguished on the basis of objective criteria (e.g., gender and age). However, when it comes to psychological characteristics, each individual in a subgroup has a certain probability of group membership and some members are more prototypical than others. Congruent with the probably nature of these group memberships, people may shift to a different group over time depending on contextual motivational supports or undermining. Hence, group membership should be seen as probable rather than determined (see for instance Gaudreau, 2013).

Second, we also encourage scholars to rely on a theory-driven approach when studying motivational profiles. Although we see it as informative for descriptive purposes to examine in an explorative way how different motivational dimensions get naturally combined into motivational profiles, the quest for such profiles becomes even more interesting when a compelling theoretical question can be addressed. To illustrate, from a multiple goal perspective (Barron & Harackiewicz, 2001), the hypothesis was developed that the most optimal outcomes would be achieved by learners who combine both mastery-approach and performance-approach goal because each of these goals would, through fairly unique pathways, yield specific benefits (Pintrich, 2000). As another example, the identification of motivational profiles can help to contrast and test predictions from more quantitative and more qualitative perspectives on motivation. If the presence of a higher amount of motivation yields more desirable outcomes, individuals combining different dimensions of motivation should function more optimally compared to those scoring high only on more qualitative aspects of motivation. If, instead, quality matters, then people characteristic of the high-quality motivation profile may display more optimal functioning than people with more characteristics of the high quantity motivation profile (e.g., Mouratidis, Vansteenkiste, Lens, Michou, & Soenens, 2013).

**The Potential of Motivational Profiling.** The more intensive study of motivational profiles has much potential for a number of reasons. First, the identification of critical profiles may appear fruitful from a diagnostic perspective. For instance, for school counselors to optimally guide students in their career decision process, it may be instructive to know whether students belong to a group that is motivationally at risk, characterized by low motivation, poor motivation, or a combination of both. The combined presence of multiple motivational deficits may put this group in an especially vulnerable position for academic maladjustment.

Second, apart from motivationally vulnerable groups, motivational profiling could also help identify a *motivationally resilient* group, including individuals who maintain adaptive functioning even under stressful circumstances (Fletcher & Sarkar, 2013) and who cope well with stressors (Skinner & Zimmer-Gembeck, 2007). This issue could be examined by first determining individuals’ membership and prototypicality of membership in different motivational profiles before exposing them to a standardized stressor in an experimental setting or before examining how they react to daily stressors in a diary design.

A third potential advantage of motivational profiling is that it may allow for a more fine-grained analysis of how the social context nurtures individuals’ engagement and motivation (e.g. De Meyer et al., 2016). Although, at least from the perspective of Self-Determination Theory, a need-supportive motivating style may enhance individuals’ engagement, well-being, and achievement across profiles, the specific manifestations of contextual need support may differ depending on individuals’ motivational profile. This identification of specific manifestations is important to gain insight in the way a motivating style needs to be tailored according to the individuals’ motivational profile. To illustrate with a speculative example, individuals in a lowly (compared to a highly) motivated group may benefit less from offered choice as lowly motivated individuals would not want to engage in the activity at all. Instead, this lowly motivated group may benefit more from an understanding and empathic approach, such that they can voice their complaints and resistance toward the activity, or may be given a meaningful rationale for their activity engagement as to create a greater readiness for activity engagement (see Mouratidis, Vansteenkiste, Lens, & Sideridis, 2011). Alternatively, some motivational scholars working from the perspective of Achievement Goal Theory (e.g., Barron & Harackiewicz, 2001) would argue that the motivational approach adopted should match or fit as good as possible with the dominant motivational orientation characteristic of a the subgroup. To illustrate, a strongly performance-oriented subgroup would then benefit from a teacher, parent or sport coach that fosters such a motivational orientation (see Mouratidis et al., 2013).

Finally, in the context of longitudinal research, motivational profiling can lead to the identification of motivational trajectories describing individuals’ development across time. In turn, these motivational trajectories can be used to predict important outcomes such as individuals’ trajectory in terms of achievement and their probability to drop out of school. Also, longitudinal studies would help to shed light on the degree of stability versus change in individuals’ profile membership and on the personal and contextual factors that impact upon individuals’ change versus stability across time (e.g., Corpus & Wormington, 2014). In sum, a longitudinal approach to motivational profiles has the potential to draw a dynamic and person-centered picture of changes in motivation and of the correlates and antecedents of these changes.

**This issue.** A number of contributions in the special issue testify to the importance of considering a variety of motives that underlie individuals’ behavior. For instance, Van der Kaap-Deeder et al. (this issue) show that adolescents high on contingent self-esteem, that is, adolescents who tend to hinge their self-worth upon achieving desirable outcomes, display various motives for putting effort in their school work. Specifically, they feel internally pressured to invest time in their school work (i.e., introjected regulation) but at the same time also more easily perceive the personal significance of putting effort in their schooling (i.e., identified regulation). Hence, their motivational functioning is characterized by an intriguing and ambivalent mix of both controlled and autonomous forms of motivation.

Taking a person-centered instead of a dimension-centered approach, (Fryer et al., this issue) sought to identify different motivational profiles in a group of high school students. They did so by relying on Willy Lens’ proposed 2x2 model, in which the distance of the aspired goal (i.e., short-term versus long-term) and the regulation underlying goal pursuit (i.e., internal-external; Lens et al., 2002) are discerned. In addition to considering these differences in the type of motivation of high school students, Fryer et al. (this issue) also considered students’ level of amotivation. Using these different dimensions, they retained through latent profile analyses three different groups, each characterized by a particular combination of motives. Overall, the group scoring highest on internal regulation and future orientation and lowest on amotivation reported the most deep-level learning.

**Summary.** In line with the layman-perspective on human motivation that our behavior is multi-determined, scholars in the field of motivation psychology have paid increasingly attention to the combinations of motives that underlie individuals’ functioning. The progress in this area was hampered for some time presumably because scholars adopted a unidimensional or bipolar view towards individuals’ type of motivation, thereby assuming that the presence of one type of motivation (e.g., intrinsic motivation; task orientation) would by definition imply the absence of another type (e.g., extrinsic motivation; ego orientation). Only when this unidimensional view was replaced by a multidimensional approach, did it become possible to identify groups of individuals characterized by specific combinations of different types motivation, thereby shedding light on the issue of motivational hetereogeneity. Although many questions are still unanswered (e.g., stability of profiles; tailoring of motivational approaches to groups), in our view this approach has potential, both at the conceptual level and at the practical level.

### Trend 3: A More Refined Insight in People’s Lack of Motivation

**Pull and Push Factors.** Motivational scholars have traditionally focused on the different factors that lead people to engage in a target activity. For instance, scholars examined the type of goals learners have in mind when doing their homework (Valle et al., 2015), the aspirations and values that employees aim to achieve via their work (Kasser, 2016; Promislo, Deckop, Giacalone, & Jurkiewicz, 2010), or the reasons patients have for sticking to their medication regime (Williams, Rodin, Ryan, Grolnick, & Deci, 1998). To the extent that individuals lack motivation for engaging in the target activity, they are said to possess low ability beliefs, low expectancies to attain desired outcomes, and to question their commitment to put effort in the activity at hand, with each of these beliefs reflecting aspects of amotivation (Legault, Green-Demers, & Pelletier, 2006). More generally, individuals’ lack of motivation is often considered a matter of lacking the necessary skills, plans, or strategies to achieve desire outcomes, such that individuals’ behavior lacks intentionality. Amotivation would then manifest through a sense of apathy, helplessness, and indifference, with individuals passively “going through the motions” (Deci & Ryan, 1985).

Yet, a host of other factors could also help to understand why individuals do not partake in a target activity. That is, much as individuals’ behavior is multi-determined, also their lack of motivation may stem from a variety of sources. The notion of *cost* within expectancy-value models (Barron & Hulleman, 2015; Eccles & Wigfield, 2002; Wigfield & Cambria, 2010) precisely conveys the idea that the overall value of a task can be impacted negatively by the perceived barriers or costs associated with performing the activity. Gaining more refined insight in these reasons for non-participation is a topic in need of further investigation (see also Barron & Hulleman, 2015). Inspired by Atkinsons’ dynamics of action (Atkinson & Birch, 1970), Willy also emphasized the importance of studying this topic to better understand why and at which point individuals’ behavior shifts from the target activity to a competing activity. For Willy, a given behavior would not occur in isolation as there is a continuous stream or flux of activities. An example of these intrapersonal motivational dynamics may illustrate Willy’s point. Consider a student who in general has an interest in doing his homework; consider him now, at some point at which he may stop doing his homework, not so much because his interest has completely waned but because the strength and type of motivation for the competing activity (e.g., leisure time; watching TV) has become more dominant. By taking into account the interplay between motives for multiple activities, we are better able to shed light on the *dynamic* nature of individuals’ behavior (Atkinson & Birch, 1966). Note that an empirical examination of these dynamic behavioral processes will ideally involve a reliance on within-person analyses rather than the classical between-person analyses generally used in psychology. Instead of focusing on relative differences between people, such within-person analyses allow for an examination of how a person’s behavior changes compared to his own behavioral trends (see Keijsers, 2016; Voelkle, Brose, Schmiedek, & Lindenberger, 2014).

In addressing the diverse reasons underlying non-participation, a distinction can be made between *pull* and *push* factors (Vansteenkiste & Soenens, 2015). Pull factors orient individuals away from the activity at hand (i.e., the target activity) toward an alternative, competing activity which has gained importance. That is, an alternative activity gets valenced as a positive outcome, thereby instigating an approach orientation (Elliot, 2008). To illustrate, a student may not make his homework because he more strongly values attending his soccer training. Thus, when pull factors are salient, people feel inclined not to partake in the target activity because a competing activity pulls for their attention.

In contrast, push factors reflect a category of motivational factors that relate primarily to the target activity itself rather than to an alternative, competing activity. In this case, people are pushed away from the target activity, which they want to escape or avoid. When push factors operate, people develop an avoidance orientation rather than an approach orientation (Elliot, 2008). For instance, a student may decide not to make his homework because he considers this refusal as a way to rebel against his teacher and against the pressure imposed upon him at school more generally. Although the direction of behavior is relatively clear in the case pull factors prompt a switch to competing activities, this is less the case for push factors. This is because individuals are avoiding a negatively valenced target behavior and because individuals do not necessarily pursue a positive alternative. Said differently, when pushed away from the target activity, it is clear what one does not want, but it is less clear what one does want (for the anti-goal gravity see Carver & Scheier, 2012).

**Differentiated Approach.** Both push and pull factors can be approached in a differentiated way. A first issue to be considered is the *type* or *content* of the alternative activity individuals engage in. In an earlier investigation regarding this topic, Lens, Lacante, Vansteenkiste, and Herrera (2005) reported that the amount of time university students spend on leisure time and work activities yielded a different relation with their academic motivation, attitude, and exam results. While the amount of time spent on work had a negative relation with these various outcomes, the relation appeared curvilinear in the case of time spent on leisure time activities. Spending between 1 and 4 hours per week on leisure time activities was the most optimal dose, whereas either no time or more than four hours spent on leisure activities being related to poorer academic outcomes.

Second, apart from the content of these competing activities, also the *type* of motives for pursuing these alternative activities deserves greater attention. From the perspective of self-determination theory, these motives could be either more autonomous or more controlled in nature. That is, people may either feel volitional and committed to engage in the alternative activity or they may feel coerced or seduced to do so. For instance, an unemployed person might decide not to search for a job because he gives priority to taking care of his sick mother or to following a vocational training. Alternatively, an unemployed person might not search for a job because his wife wants him to take care of the children or because he feels like a bad father if he would not be sufficiently involved in his children’s lives. Different from amotivation, which is characterized by low intentionality, autonomous and controlled participation in the competing activity are intentional (i.e., oriented toward a specific outcome).

Note that this differentiation between qualitatively different types of reasons for not partaking in the target activity deviates from how the notion of cost has typically been conceived in expectancy-value models (Barron & Hulleman, 2015; Eccles & Wigfield, 2002). Typically, a variety of costs (e.g., amount of time needed to perform the target activity; loss of time to spend on valued alternative activities) are summed in an overall ‘cost’ index. We deem it worthwhile to adopt a more differentiated approach to the notion of cost, thereby examining how different motives for not participating in a target activity and instead participating in an alternative activity relates to individuals’ functioning.

Illustrative in this context is a study by Willy and colleagues, who studied unemployed people’s reasons to both search and not search for a job (Vansteenkiste, Simons, Lens, Sheldon, & Deci, 2004). In addition to being asked why they were searching for a job, long-term unemployed individuals were asked why they were not searching, thereby tapping into their autonomous and controlled motives not-to-search. Interestingly, the motives not-to-search predicted additional variance in participants’ unemployment experiences and general well-being above and beyond the “classic” SDT-based motives to search (i.e., autonomous motivation, controlled motivation, and amotivation). Specifically, controlled motivation not-to-search yielded a unique positive relation to a negative experience of one’s unemployment. Autonomous motivation not-to-search related positively to a positive unemployment experience and to overall well-being. Whereas autonomous motivation to search was unrelated to general well-being, the often observed positive correlates for autonomous motivation were found for the construct of autonomous motivation not-to-search. Presumably, unemployment allows autonomously non-engaged individuals to achieve their ideals and values, while autonomously job seeking individuals have not yet achieved their personally valued goal of being employed (see also Halvari, Vansteenkiste, Brorby, & Karlsen, 2013). Further, we would predict the type of motives underlying the competing, alternative activity may predict individuals’ self-regulation impairment. To illustrate, if students have foregone studying in favor of engaging in a leisure activity for controlled (e.g., attending the soccer training is an obligation) instead of autonomous (e.g., they like playing soccer), they may experience more self-regulation impairment and more motivational interference during and more regret after terminating the leisure activity (see Grund, Brassler, & Fries, 2014; Kuhnle, Sinclair, Hofer, & Kilian, 2014).

In analogy with the observation that individuals can display different motives for partaking in a competing activity, they may also have different reasons for foregoing the target activity. That is, their reluctance to engage in the target activity could be either more controlled or more autonomous in nature. When controlled, their refusal involves a reactive opposition against the target activity as individuals take distance from pressuring external of internal forces (Brehm, 1966; Koestner & Losier, 1996). That is, their controlled non-participation stands in the service of gaining independence. This search for independence, however, is driven by pressured and internally conflicted motives as individuals directly oppose the pressuring forces they encounter. These forces can be external or internal in nature. To illustrate, a drug addict could avoid seeking help to save face in front of her in-group (i.e., external pressure) or because she feels ashamed and anxious to disappoint her parents if she admits having a drug problem (i.e., internal pressure). Interestingly, although such controlled non-participation is often oriented toward the restoration of lost freedom, recent research indicates that it does not contribute to enhanced volitional functioning. On the contrary, it has been found to increase interpersonal distance and alienation (Van Petegem, Vansteenkiste, Soenens, Beyers, & Aelterman, 2015), which is probably due to the fact that individuals high on controlled non-participation fail to pursue their personal interests, values, and commitments (Koestner & Losier, 1996).

In the case of autonomous non-participation, individuals abstain from engaging in the target activity after considerable reflection, such that they do not partake in the target activity more willingly. That is, they have made a more informed decision not to participate in the activity. Different from amotivation, both autonomous and controlled non-participation are intentional in nature as both are oriented toward a specific goal (i.e., abstaining from the target activity). Yet, controlled non-participation represents a more blunt and defensive form of non-participation, as individuals directly oppose the requests imposed upon them. That is, it reflects a form of ‘anti-internalisation’ (Aelterman et al., 2016). Instead, the non-participation would be more selective and well thought-out in the case of autonomous non-participation. After introspection, individuals would have come to the conclusion to be unwilling to engage in the activity and to stay true to themselves by not engaging in the activity.

Research on these different forms of defiance is limited. In one recent study among students in physical education classes (Aelterman et al., 2016), controlled reasons for not putting effort in the PE class could be distinguished through factor analysis from other motives and yielded a unique relation in the prediction of resentment vis-à-vis both the content of the lesson as well as the teacher. Clearly, more work is needed in this area, as this topic has the potential to provide a more refined and nuanced insight in people’s motivational functioning, an issue we elaborate upon next.

**Potential.** The examination of both push and pull factors for not engaging in a target activity deserves more in-depth attention, as such an examination may enrich insight in motivational dynamics in various ways. First, research on motives for non-participation may add a developmental perspective to motivational research. There are likely age-bounded shifts in the various reasons for not partaking in an activity. Research by Kuczynski en Kochanska (1990) suggests that toddlers gradually learn to replace a crude form of resistance against maternal requests with a more constructive type of non-participation characterized by dialogue and negotiation to express their disagreement. As children learn to cope differently with demands (Skinner & Zimmer-Gembeck, 2007), their reasons for not engaging in the target activity may also change from being less oppositional and controlled toward more reflective and autonomous.

Second, the study of people’s reasons for not partaking in an activity allows one to more strongly connect existing motivational frameworks in the field of education, sport and work psychology with clinical models (e.g., Motivational interviewing), social psychological frameworks (e.g., psychological reactance theory) and the identity literature (e.g., Erikson, Marcia). Miller and Rollnick (2002, 2012) precisely developed Motivational Interviewing such that clinicians and health care providers could better handle the resistance displayed by their clients. That is, the discord between clinicians and clients is seen as being function of a confronting and pressuring approach, while an empathic approach is considered critical to take clients’ resistance seriously. Similarly, Brehm (1966) argued that individuals’ psychological reactance is rooted in a loss of freedom and serves a restorative purpose. The study of people’s autonomous reasons for partaking in an alternative activity may help to shed light on the functioning of individuals’ inner compass (Assor, 2012), which reflects individuals’ emerging interests, personally endorsed values, and long-term aspirations. To the extent that individuals’ internal compass is fully developed and operating smoothly, people may have the capacitity to regulate their ongoing activities in an autonomous fashion, thereby being able to develop priorities and to shift from a target to a competing activity for autonomous reasons.

Third, the motivational profiling work described in the preceding paragraph could be extended, thereby shedding light on how groups of individuals combine reasons for partaking and reasons for not partaking in an activity. A motivationally vulnerable group may be one where individuals feel discouraged to partake in the activity (i.e., amotivation), react against the pressure imposed upon them to prove their independence (i.e., controlled non-participation), while at the same feel that they still should engage in the target activity (i.e., controlled participation). Such individuals may experience a lot of inner conflict and ambivalence, oscillating between partaking and foregoing the activity.

**This issue.** In the present issue, Chen et al. conducted a vignette-based study to examine whether participants’ oppositional defiance against a maternal request to study more would vary as a function of the style being used by the mother. Oppositional defiance was conceived as a maladaptive coping response, next to compulsive compliance, that is, the tendency to strictly obey imposed requests out of a sense of obligation (Skinner & Zimmber-Gembeck, 2007). In case the mother was depicted as using a controlling style in the vignette, participants were more likely to defy the request to put extra effort in their studies, while also reporting more compulsive compliance relative to when the same request was presented in a more autonomy-supportive way. Thus, pressure elicited an intriguing mix of feeling forced to partake in the activity, while at the same time desiring to escape the request all together. Future work could tap into the reasons for defiance or compliance, thereby trying to shed light on the motivational ambiguities underlying much of our behavior.

**Summary.** To summarize, scholars in the field of motivation psychology have increasingly paid attention to individuals’ motives for not engaging in a target activity. Oftentimes people perceive a variety of costs being associated with a target activity (Eccles & Wigfield, 2002). From our perspective, these costs can best be approached in a differentiated way, as individuals sometimes get pulled into a competing activity and at other times feel pushed away from the target activity. Both the inclination to be pulled toward alternative activities and the inclination to be pushed away from a target activity can be driven by a variety of reasons, with some of these reasons being more volitional and autonomous and with other reasons being more pressured, conflicted, and controlled.

### Trend 4: Towards a Nuanced Perspective: On Motivational Universalism and Relativism

**Motivational Universalism and Relativism.** One final theme that has attracted increasing attention in the field of motivation psychology is the extent to which motivational dynamics are universally applicable or instead depend on a number of constraining factors. A variety of factors have been proposed and studied in this respect. A first set of factors include objective personal and socio-demographic characteristics, such as gender (Katz, 2016; Lietaert, Roorda, Laevers, Verschueren, & De Fraine, 2015) socio-economic status (e.g., Snibbe & Markus, 2005), and age (e.g., Vecchione, Alessandri, & Marsicano, 2014). Another set of factors encompasses more psychological characteristics that lie at the personal level, as reflected in personality differences (e.g., Rietzscehl, Stijkhuis, & Van Yperen, 2014), or at the contextual level, as reflected in differences in the more proximal environment (e.g., perceptions of the climate in class, in the family, at work; DeMeyer et al., 2016) and in the broader environment (e.g., socio-cultural climate; e.g., Markus & Kitayama, 2003).

Presumably, scholars’ position regarding these issues can vary between two extremes, with the one pole representing an extreme universalistic position and with the other pole reflecting an extreme relativistic position (Soenens, Vansteenkiste, & Van Petegem, 2015). From an extreme universal perspective, the same motivational dynamics would apply to all individuals, regardless of interindividual and contextual differences. From an extreme relativistic perspective, it would be argued that it is useless to discuss general motivational processes because these processes are strongly determined by a long list of complexly interacting determinants. In the end, motivational processes would be almost idiosyncratic and unique to each individual, such that any type of generalization is unwarranted. Although only few scholars might adopt such extreme views, there likely exists quite some variability in scholars’ exact position along this continuum.

To illustrate, although being performance-oriented is generally said to yield fewer benefits compared to being task-oriented from the normative goal perspective (Midgley et al., 2001), scholars have maintained and empirically examined whether a performance orientation may possibly come with greater advantages if one finds oneself in an environment that promotes such an orientation (Barron & Harackiewicz, 2003; Lens, personal communication). Within such an environment, one’s personally held achievement goals would fit with the goals emphasized by the social context, creating a matching situation. The available evidence in favor of the match-hypothesis is mixed, with some studies (e.g., Murayama & Elliot, 2009) providing some evidence for a a beneficial effect and others (e.g., Linnenbrink, 2005) failing do so. In his own work, Willy also addressed this issue, thereby examining whether the contribution of individuals’ life goals in predicting effort-expenditure, test anxiety and achievement would depend on the type of life goals being promoted by adolescents’ parents (Mouratidis et al., 2013). In this study, no evidence was for the advantages of a matching situation (see also Dittmar, Bond, Hurst, & Kasser, 2014).

As another illustration, the question whether the psychological needs identified in Self-Determination Theory yield universal benefits is a topic of debate and research. As these psychological needs are said to be evolutionary evolved, they are part of individuals’ psychological make-up from the very beginning in their lives. Given this fairly strong position, several scholars have examined and some have questioned the claimed universal benefits of need satisfaction, especially with respect to the most controversial need, that is, the need for autonomy (Ryan & Deci, 2006). To address the potentially constraining or amplifying role of sociodemographic and psychological characteristics a number of considerations need to be taken into account.

**Considerations.** We want to highlight three issues that deserve attention when addressing questions regarding universality of motivational effects. First, it seems critical not to treat mean-level differences in motivational constructs as evidence in favor of a relativistic account because mean-level differences do not speak to the question whether associations of motivational constructs with antecedents and outcomes differ across groups. For instance, girls have often been found to be more autonomously motivated (e.g., de Bilde, Vansteenkiste, & Lens, 2011; Katz, 2016), while boys tend to be more ego-oriented (e.g., Chin, Khoo, & Low, 2012). Yet, such mean level differences do not imply that boys and girls would benefit to different degrees from being autonomously motivated or ego-oriented. Similarly, Chinese learners have typically experienced their parents and teachers to be more pressuring and controlling compared to Western learners, including for instance Belgian (e.g., Wuyts, Chen, Vansteenkiste, & Soenens, 2015) and American students (Pomerantz & Wang, 2009). Yet, the elevated pressure that is experienced by Chinese learners does not make them immune for its negative effects, as if the normative occurrence of control within the environment would completely offset its detrimental impact (Gershoff et al., 2010).

Indeed, there is a logical leap between the observation of mean-level differences in motivational constructs as a function of sociodemographic and psychological characteristics and the claim that these characteristics alter the functional role of the motivational constructs. The structural effects of key motivational pathways and approaches cannot be inferred on the basis of mean-level differences. To examine the effect of sociodemographic and psychological characteristics on the functional role of motivation, researchers need to investigate the extent to which these characteristics change (i.e., moderate) the predictive validity of motivational variables (e.g., Chen et al., 2015; Lietaert et al., 2015). Importantly, to adequately address this issue, the items tapping into these motivational constructs need to carry a similar meaning across the studied subgroups. If the attributed meaning of the item varies across age, gender, or cultural background, it is important to understand why this is the case before proceeding with an examination of associations between motivation and other constructs in different groups.

Second, when addressing the role of these potentially moderating factors, it seems critical to differentiate between factors that affect the strength of the effect of motivational variables and factors that fundamentally alter its effectiveness. While the former type of moderation is a matter of gradation and technically concerns an ordinal interaction, the latter type of moderation is a matter of cancelling out an effect or even reversing it, which technically manifests as a cross-over interaction. From an extreme relativistic perspective, cross-over interactions should emerge as the motivational ingredients that work for one group of individuals would yield no effect or even an opposite effect for another group of individuals. From an extreme universalistic perspective, no interactions should emerge whatsoever, as the effects of motivational pathways and approaches would be invariant and applicable across persons and situations. In practice, cross-over interactions are less common than ordinal interactions, suggesting that effects are more a matter of gradation. For instance, it has been shown that individuals scoring higher on the motive to succeed (Atkinson & Feather, 1966) benefit somewhat more from competence satisfaction (Schüler, Sheldon, & Fröhlich, 2010). Such findings are inconsistent both with the extreme universalist and with the extreme relativistic position and suggest that people differ in their sensitivity to motivational effects. It would be inaccurate, however, to conclude on the basis of the finding in this example that the need for competence is not important to everyone. It is important to everyone, but to different degrees.

Related to this, there is increasing interest in the question whether there exist interindividual differences in need strength, with these differences possibly affecting the effectiveness of the satisfaction of these needs. This question was also of great interest to Willy. Being trained by John Atkinson as a post-doc scholar, Willy sought to reconcile his view on interindividual differences in need strength with the universality claim of SDT. He co-authored a contribution (Chen et al., 2015), which only appeared after his death, showing that need strength failed to moderate the association between experienced need satisfaction and well-being/ill-being. Instead, the well-being enhancing role of need satisfaction applied to all individuals regardless of how much they explicitly valued or desired getting their psychological needs met and regardless of the nation they were living in (i.e., Belgium, US, China, & Peru), with these nations differing substantially in cultural background (Chen et al., 2015). Other studies (e.g., Schüler et al., 2010), making use of different measures of need strength (e.g., implicit) and including domain-specific instead of global outcomes do provide some evidence for the moderating role of need strength. Yet, also in these studies, the observed effect is often one of gradation, with the effect of need satisfaction being less pronounced among those scoring lower on implicit need strength measures.

Third, we suggest differentiating between the actual motivational approach given by a socializing agent (e.g., coach, teacher, parent) and the way how this motivating approach is appraised (Soenens et al., 2015). While a teacher may want to promote a task-orientation and devalue a performance-orientation, students may not necessarily perceive the teacher as such. Although the actual and perceived motivating approach may be related, this relation is far from perfect. Indeed, in many cases, there exist substantial discrepancies between socializing agents’ intended and believed motivational approach, their actual motivating style, and the way the motivating style is perceived by individuals on the receiving end. The question then needs to be addressed which factors can explain the size of this discrepancy. Some of the characteristics highlighted by scholars working from a more relativistic approach may be involved (e.g., with perfectionist children more easily perceiving parental behavior as being controlling). Yet, when the actual motivating practices are perceived to be motivating, one would expect them to yield a motivating effect for all people, regardless of differences in objective background or psychological characteristics. Said differently, although people differ in their sensitivity to pick up and perceive potentially motivating behaviors by socializing agent, once the socializing agent is perceived to be motivating it is likely that there will be benefits for everyone. In this respect, proponents of the relativistic and universalistic position are both right to some extent. However, they emphasize different parts in the chain of motivational events, with universalists highlighting the presumed universal benefits of individuals’ inner motivational experiences (i.e., ‘output side’ of the chain) and with relativistic scholars highlighting the fact that there is variability in how contextual factors feed into individuals’ inner motivational experiences (i.e., ‘input side’ of the chain).

**This issue.** Several contributions in this special issue fit within this trend in the literature. Two sets of findings deserve being highlighted, dealing with (a) mean level (dis)similarities in motivational variables and (b) (dis)similarities in the contribution of these motivational variables in the prediction of outcomes. First, regarding mean level differences in motivational functioning, several authors reported differences in the studied motivational variables according to gender (Fryer et al., this issue; Michou et al., this issue; Van der Kaap-Deeder et al., this issue), age (Delrue et al., this issue) and culture (Chen et al., this issue).

Second, Cordeiro, Paixao, Lens, and Lacante (this issue) provided validity and reliability evidence for the Portuguese version of the Basic Psychological Need Satisfaction and Need Frustration Scale (BPNSF; Chen et al., 2015), thereby showing that need frustration predicts increases in ill-being over a 9-month period. In addition, Michou et al. (this issue) found need frustration to account for the negative relation between fear of failure and pursuing mastery goals for controlling reasons. Although these two contributions did not directly address the issue of moderation as no contrasting sample was involved, Chen, Soenens, Vansteenkiste, Van Petegem, and Beyers (this issue) directly tackled this topic. They addressed the question whether Belgian and Chinese adolescents make different appraisals of parental statements reflecting guilt-induction, harsh control, and autonomy-support. Adolescents were presented with different vignettes in which the mother requested the child to put extra effort in its studies after a poor grade, thereby manipulating the style of introducing the request. While the harshly controlling approach was interpreted as equally pressuring among both Chinese and Belgian adolescents, the guilt-inducing approach carried a less pressuring connotation for the Chinese adolescents. Even among Chinese adolescents, however, it was still perceived as more pressuring than the autonomy-supportive approach.

Finally, focusing on variation in individuals’ vulnerability for contingent self-esteem instead of cultural variation, Van der Kaap-Deeder et al. (this issue) examined whether individuals scoring high on contingent self-esteem would display a stronger motivational backdrop after receiving experimentally provided negative feedback. This was not the case, as the provision of negative feedback and contingent self-esteem were uniquely and independently related to greater tension during the puzzle solving activity and less free-choice behavior afterwards. Overall, the pattern of findings seems more in favour of the universalistic approach, yet, interesting mean-level differences have been obtained (e.g., Chen et al., this issue). As this field is rather in its infancy, more research is called for detailing the universalistic and more group-specific processes in motivational functioning.

### Conclusion

The field of motivation psychology is “alive and kicking”, perhaps more than it has ever been (Ryan, 2012a). Willy would have found truly exciting to notice that so many scholars and practitioners are concerned with the question which factors lead people to initiate and persist in their behavior and how individuals can be motivated in sustainable ways. Undoubtedly, Willy has had a significant role in putting this topic so high on the research agenda. He has intrigued, inspired, and ultimately even paved the way for many of us, not only in Flanders, Belgium, his home country, but across the world. Indeed, this special issue brings these scholars together from all continents and aims to further address a number of emerging trends in the field. Much as Willy shared his enthusiasm with other scholars about concepts, findings, and achievements, we want to share our enthusiasm regarding this special issue with the readership of *Psychologica Belgica.* We hope that this opening contribution and the various separate empirical contributions may further stimulate the debate and empirical research about motivational dynamics. The special issue closes with a personal tribute to Willy, discussing his personality, interests and way of being in the academic community.

## Footnotes

But even if poor and good quality types of motivation are assessed separately, the subtraction of the one score from the other to obtain a single composite score precludes one to study whether both poor and good quality types of motivation co-exist.
